# Kinetin and arbuscular mycorrhizal fungi: vital regulators of *Vicia faba* plantsʼ response and tolerance to drought stress

**DOI:** 10.1186/s12870-025-07260-9

**Published:** 2025-08-29

**Authors:** Reda E. Abdelhameed, Hanan Gahin, Rabab A. Metwally

**Affiliations:** Botany and Microbiology Department, Faculty of Science, Zagazig University, Zagazig, 44519 Egypt

**Keywords:** Kinetin, Endophytes, Drought, Legume, Peroxidation, Osmolytes, Glomalin

## Abstract

In light of the detrimental consequences of climate change and global warming, drought (water deficit) has emerged as a major abiotic stressor that adversely affects plant development, productivity, and sustainable agriculture globally. *Vicia faba* L. (faba bean), a highly nutritious leguminous crop, is especially vulnerable to water scarcity. As a possible solution, this study highlighted the recent advances in plant stress physiology regarding the role of kinetin (20 mg/L) and arbuscular mycorrhizal (AM) fungi in enhancing *V. faba* resilience to drought (30% water holding capacity) with emphasis on their growth, physiological and biochemical mechanisms. Under controlled conditions, drought markedly decreased plant growth, photosynthetic pigments (chlorophyll a + b and total pigments), and relative water content (RWC), while increasing stress markers (hydrogen peroxide and electrolyte leakage). Nevertheless, these negative effects were considerably lessened by AM fungi and kinetin application. Their application led to the improvement of *V. faba* growth parameters, maintaining cellular hydration (high RWC), higher activity of antioxidant enzymes (superoxide dismutase, catalase, peroxidase, ascorbate peroxidase, and polyphenol oxidase) and organic adjustments which include total soluble protein, proline and total soluble carbohydrate. The most surpassing effect is that AM fungal inoculation enhanced the soil-rich glomalin content, both easily and total extractable. Regarding the effect of drought stress on mycorrhizal colonization; microscopic observation showed a noticeable reduction in the formation of arbuscules and vesicles under drought. Although reduced colonization, AM fungi can nevertheless benefit host plants. These findings highlight the potential of integrating AM fungal inoculation or kinetin treatment as an eco-friendly strategy to enhance drought resilience in *V. faba* cultivation.

## Introduction

In natural environments, plants are constantly encountering fluctuations in environmental cues [1]. It is anticipated that future global climate change will accelerate due to the ongoing increase in air temperature and atmospheric CO_2_ levels, which eventually change the distribution and patterns of rainfall [2, 3]. The primary cause of drought or water deficit stress is typically inadequate rainfall-induced water intake, elevated temperatures, intense light, and dry winds can cause evaporation, which further exacerbates an already-existing drought stress event [4, 5]. On a worldwide scale, drought significantly limits agricultural development especially in arid and semi-arid regions as it has a multifaceted effect on plants, affecting plant phenotype, physiology, and biochemistry [6]. It adversely affects plant growth and development by reducing water availability, impairing nutrient uptake, and disturbing metabolic and physiological processes and leads to the buildup of reactive oxygen species (ROS), ultimately reducing yield and plant survival [7, 8]. Egypt, as an arid country, is one of several nations with water shortage issues, which have recently gotten worse as a result of limited supplies, the country’s rapid population growth and fixed water allocation from the Nile River [9, 10].

Leguminous crops like *Vicia faba* L. are crucial for sustainable agriculture due to their high nutritional value and nitrogen-fixing facilities, although their sensitivity to water deficit during growth stages renders them vulnerable to yield losses under drought circumstances [8, 11]. So, their sensitivity to drought necessitates effective mitigation strategies. Traditional breeding methods have had limited success in developing drought-resistant varieties. Developing alternative and effective strategies to mitigate the detrimental effects of drought in *V. faba* is critical to ensuring food security and sustainable agriculture, especially in drought-prone regions. Among these effective strategies, the application of phytohormones such as kinetin and the utilization of endophytic fungi have emerged as promising approaches to enhance drought tolerance [12, 13].

Cytokinins, particularly kinetin, play a significant role in regulating various physiological and biochemical processes in plants, including cell division, chloroplast development, senescence delay, and stress tolerance [14, 15]. A study by Abeed et al. [16] stated that the application of kinetin triggered a clear shift from downregulation to upregulation of all drought tolerance traits in wheat cultivars by redirecting photoassimilates toward vegetative sinks which led to enhanced growth, increased accumulation of osmoregulatory compounds, improved tissue vigor and morphological adjustments. Additionally, under drought conditions, kinetin modulates the expression of stress-responsive genes and maintains cellular homeostasis, contributing to improved water status, photosynthetic efficiency, and overall plant resilience [17]. It sustains stomatal conductance, enhances osmotic adjustment, and boosts antioxidant enzyme activity, such as superoxide dismutase (SOD), catalase (CAT), and peroxidase (POX), which play vital roles in detoxifying ROS generated during stress. The exogenous application of kinetin improved plant resistance to drought stress by enhancing growth and various physiological processes which are negatively affected by drought stress [18, 19].

Among other effective techniques, symbiotic connection with arbuscular mycorrhizal (AM) fungi, a common type of soil microbe, can form adaptive mechanisms with their host plants and form symbiotic associations with the roots of more than 80% of plant species [20–23]. By expanding root surface area, promoting nutrient and water uptake, and modifying hormonal and antioxidant pathways, mycorrhiza enhances plant performance in water-limited environments [24–27].

A study by Singh and Singh [26] reported that AM fungi improved the nutritional status and leaf relative water content (RWC) in tomato plants, facilitating more efficient mineral translocation and mitigating the adverse effects of drought on plant growth. In legumes, mycorrhizal symbiosis has been linked to improved nitrogen fixation, enhanced root development, and greater drought tolerance [28]. Soliman et al. [13] and Kakouridis et al. [29] discovered that AM fungi can help plants respond less negatively to water stress by acting as extensions of the root system along the soil-plant-air water flow continuum. In *Populus cathayana*, Han et al. [30] revealed AM-induced genes that mostly enhanced the antioxidant enzyme system and osmotic control, hence contributing to drought stress resistance. According to Liu et al. [31], under stress, AM fungi changed a number of pathways linked to the metabolism of organic acids and amino acids in peanut roots while maintaining the architecture of mitochondria and chloroplast thylakoids.

Despite the documented benefits of kinetin and AM fungi in different crops, detailed studies evaluating its physiological and biochemical impacts on *V. faba* plants under drought stress remain limited. Therefore, this study aims to evaluate the individual role of kinetin and mycorrhizal fungi in improving drought stress tolerance in *V. faba* plants. By assessing morphological, physiological, and biochemical responses, this research donates to a better understanding of how biostimulants can enhance crop resilience in water-lacking environments.

## Materials and methods

### Plant material and growth conditions

Seeds of *Vicia faba* L. (broad bean: Sakha 3) were supplied by Crop Institute, Agriculture Research Centre, Ministry of Agriculture, Giza, Egypt. The seeds were surface-sterilized using 30% sodium hypochlorite for 5 min, rinsed thoroughly with distilled water, and sown in plastic black bags (17 cm in diameter and 20 cm in height) filled with a sterilized 4 kg soil: clay to sand mixture (2:1). This soil was previously autoclaved at 121 °C for 1 h on three consecutive days to eliminate native microorganisms, including mycorrhizal propagules [32]. This work was carried out in the Botany and Microbiology Department, Faculty of science, Zagazig University during the winter season of 2024/2025. Plants were grown under conditions with a 14/10 h light/dark photoperiod, temperature of 24 ± 2 °C, and relative humidity of 60–70%.

**For mycorrhizal inoculation**, the AM fungal inoculum used in this study was *Rhizophagus irregularis*,* Gigaspora margarita*,* Funneliformis mosseae* and *F. constrictum*, Each pot received 50 g of inoculum containing spores, hyphal fragments, and infected root pieces at sowing time. The inoculum was applied directly below the seeds to ensure effective colonization. For control (non-mycorrhizal) treatments, a filtrate (25 μm mesh) of the inoculum was added to provide the same microbial wash (minus AM fungal propagules) to normalize microbial exposure [24].

Regular irrigation was maintained until drought stress was imposed. After 1 week of cultivation, uniform seedlings were selected 5 plants/pot and pots were laid out in a completely randomized design with the following factorial six treatments with five replicates per treatment (5 × 6) giving total of 30 pots:

1. **Control (C)**: Well-watered: Plants were irrigated with 90% water holding capacity (WHC).
2. **Drought stress (DS: 30% WHC)**: Plants were irrigated with 30% WHC.
3. **Kinetin (KN)**: Plants were foliar-sprayed with kinetin.
4. **AM**: Plants were inoculated with arbuscular mycorrhizal inoculum.
5. **Drought + Kinetin (DS + KN)**: Plants were irrigated with 30% WHC + foliar-sprayed with kinetin.
6. **Drought + AM (DS + AM)**: Plants were irrigated with 30% WHC + Inoculation with arbuscular mycorrhizal inoculum.

**Implementation of drought and kinetin treatments**: Drought stress was initiated three weeks after germination by adjusting the soil water capacity at (30% WHC) using gravimetric methods. The stress period continued for 3 weeks. For kinetin application, kinetin (6-furfurylaminopurine) was supplied from SIGMA and applied as a foliar spray at a concentration of 20 mg/L, prepared in distilled water and applied thrice: at the onset of drought and two times later. Each plant was sprayed with 25 mL of kinetin solution. After the drought period, sampling was done following a thorough cleaning of all plants from each treatment with distilled water and a gentle paper towel to evaluate the growth traits, and physiological and biochemical determinations.

### Measured parameters

#### Mycorrhizal colonization

Following the method of Phillips and Hayman [32], root samples of *V. faba* were cleared in 10% KOH and stained with 0.05% trypan blue to notice AM fungal structures under a microscope, besides hyphal (HC), vesicular (VC) and arbuscular colonization (ARC) (%) were estimated.

#### Growth and biomass

Growth related parameters, such as plant height (cm), leaf number, and total fresh/dry biomass (g) were recorded at sampling. Dry biomass was obtained after oven-drying samples at 70 °C for 72 h.

#### Physiological measurements

Relative water content (RWC) was determined following the method of Barrs and Weatherley [33] and calculated based on the following formulae: RWC (%) = [(Fresh weight – Dry weight)/(Turgid weight – Dry weight)]*100. The leaf RWC was estimated by recording the fresh weight (g) of leaf samples, thereafter immediately dipped in Petri dishes containing distilled water for 4 h to record turgid weight (g), followed by drying in a hot air oven at 70 °C till constant dry weight (g) reached.

Chlorophyll content was measured spectrophotometrically in 80% acetone extracts by using double beam spectrophotometer (RIGOL, Model Ultra-3660). 100 mg of leaf sample was collected in a bottle containing 20 mL acetone and the absorbance of the supernatants was read at 645, 452.5 and 663 nm using 80% acetone as blank [34].

### Electrolyte leakage (EL) and hydrogen peroxide (H_2_O_2_) content

The Lutts et al. [35] approach was used to measure EL or the drought injury index. In short, 20 leaf discs from unstressed and drought-stressed plants were cleaned using distilled water. After that, the disc samples were left at room temperature in 20 mL of distilled water. After two hours, a conductivity meter was used to measure the solutions’ initial conductivity (ELi). The samples were heated to 120 °C for 20 min in a water bath and cooled to room temperature, and the final conductivity (ELf) of the resultant solutions was then measured. This formula was used to determine the EL: EL (%) = (ELi/ELf) × 100.

A spectrophotometer was used to measure the H_2_O_2_ level by Velikova et al. [36]’s procedure. 500 mg of fresh leaf tissues were homogenized using trichloroacetic acid. For 15 min, the homogenate was centrifuged at 8000 g. Then, 0.5 mL of the supernatant was combined with 2 mL of potassium iodide (1 M) and 0.5 mL of 10 mM potassium phosphate buffer (pH 7.0). Each sample’s H_2_O_2_ content was calculated as mg/g Fw by comparing its absorbance at 390 nm to a standard calibration curve.

### Determination of organic osmolytes (proline, protein and carbohydrates)

The Bates et al. [37] method and a standard graph with a range of proline concentrations were used to compute the proline content. After crushing 500 mg of fresh leaf samples in a 3% sulfosalicylic acid solution, the samples were allowed to settle for five minutes. The supernatant was mixed with glacial acetic acid solution and acid ninhydrin (1:1:1) after centrifugation at 10,000 g. The mixture was heated to 100 °C for 60 min and then chilled in an ice bath. To test the absorbance of the resultant solution at 520 nm, toluene was employed as a blank to collect the chromophore.

The phenol-sulfuric acid technique [38] was used to quantify the total soluble carbohydrates that were extracted from dry leaf tissue using HCl. In accordance with Lowry et al. [39], the total soluble protein was extracted in phosphate buffer (pH 7.0) and quantified by combining 1 mL of extract with 5 mL of sol C (50 mL sol A {2% Na_2_CO_3_ in 0.4% NaOH} +1mL sol B {0.5% CuSO_4_ in 1% sodium tartrate}), shaking thoroughly, and then letting it sit at room temperature for 10 min. After that, 0.5 mL of Folin reagent was added, thoroughly shaken, and left at room temperature for 30 min before the absorbance at 750 nm was measured. The protein content was measured in mg of Bovine Serum Albumin equivalent per gram of fresh weight.

### Glomalin content

The methods for measuring glomalin content in soil, including easily extractable (EEG) and total glomalin (TG), comprise specific extraction and quantification techniques [40]. To measure EEG content, soil samples are first exposed to an extraction process using a mild buffer solution, typically 0.01 M citrate (pH 7.0). The soil is heated at approximately 60 °C for 30 min, after which the mixture is centrifuged to separate the glomalin from the soil particles and the extracted solution is then scrutinized for protein content [39]. Concerning TG content, on the other hand, includes both EEG and glomalin more tightly bound to soil particles. This is extracted through a more intense procedure, where the soil is heated under high pressure with 50 mM sodium hydroxide at 121 °C (autoclaving). The slurry is then centrifuged, and the TG is measured similarly to EEG.

### Antioxidant enzymatic activity assays

Fresh leaf samples weighing 1 g from each treatment were homogenized in 10 mL of ice-cold extraction solution that contained 1% (w/v) polyvinylpyrrolidone, 0.1 mM ethylenediaminetetraacetic acid (EDTA), and 50 mM potassium phosphate buffer (pH 7). At 4 °C, the homogenate was centrifuged for 20 min at 8000 g. The enzymatic tests were performed using the obtained supernatant. The reduction method of nitro blue tetrazolium was used to evaluate SOD activity [41]. In an aluminium foil-lined box with two fluorescent lamps at 25 °C, test tubes holding reaction solution comprising 3 mL of assay buffer, 60 µL of crude enzyme, and 30 µL of riboflavin were lit for seven minutes. Following the reaction, a spectrophotometer was used to measure the absorbance of the reaction solution and blank solution at 560 nm. The following formula was used to determine SOD activity:

$$\text{SOD activity}\:(\%)=\:(1-A/B)\times100$$

Sample absorbance is denoted by A, and blank absorbance by B.

Briefly the activities of catalase (CAT: EC 1.11.1.6), peroxidase (POX: EC 1.11.1.7): polyphenol oxidase (PPO: EC 1.14.18.1) and ascorbate peroxidase (APX: EC 1.1.11.1) were measured in the supernatant and expressed as units/g fresh weight following the protocol of Aebi [42], Maehly and Chance [43], Zhan et al. [44] and Nakano andAsada [45], respectively.

### Statistical analysis

The mean ± standard error (SE) of five biological replicates is used to express the results. Using SPSS software (version 20), data were analyzed using one-way ANOVA and Duncan test to identify significant differences (*p <* 0.05) among treatments.

## Results and discussion

### Mycorrhizal colonization and symbiotic efficiency(Plant-fungi interaction)

**Fig. 1:**
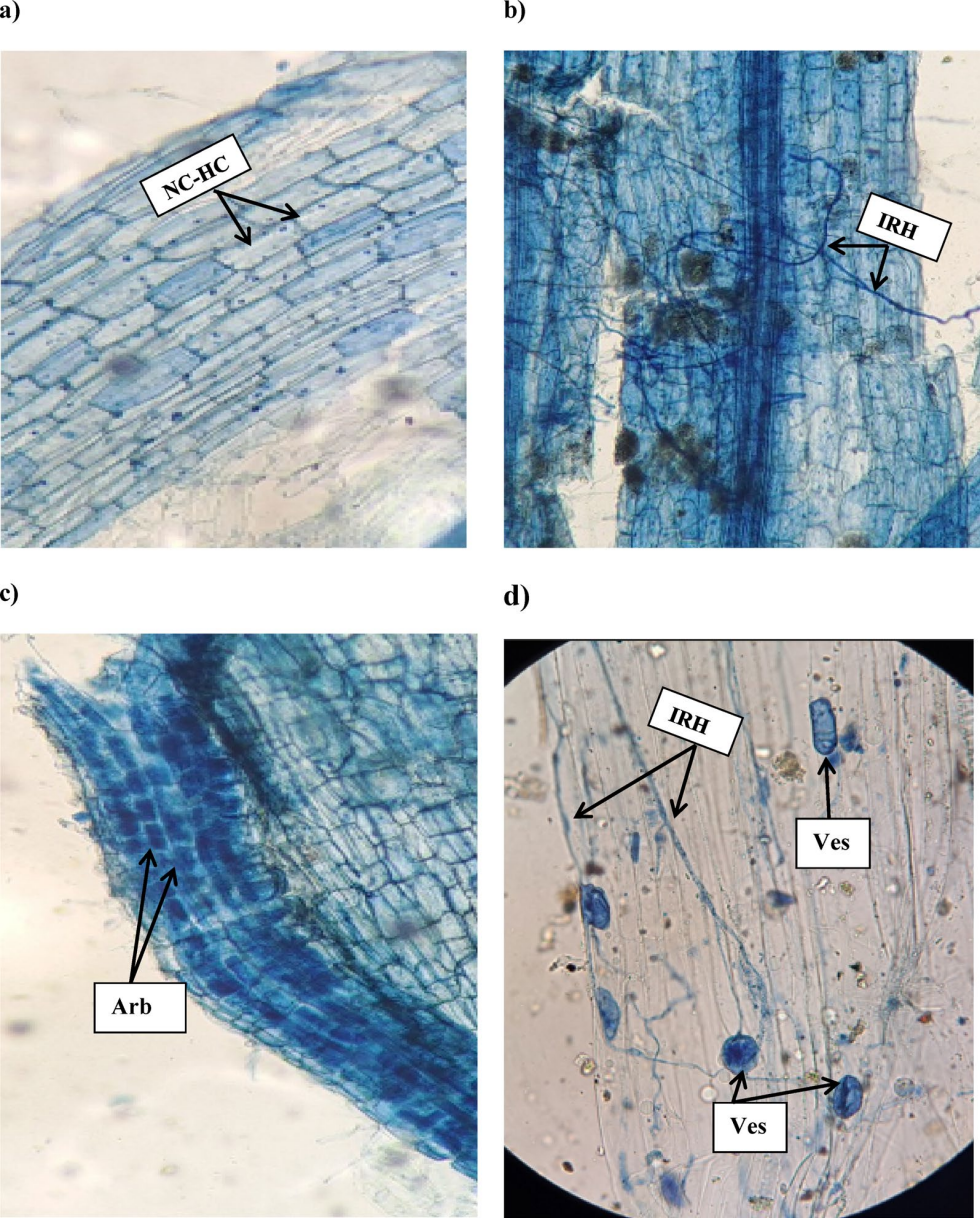
Symbiotic efficiency between mycorrhizal fungi and *V. faba* roots showed different mycorrhizal structures (hyphae, arbuscules and vesicles) using light microscope (10×). Control non-inoculated *V. faba* roots (**a**). Structures representative of AM (**b**,** c **and** d**) using these abbreviations: NC-HC (Non-colonized Host cells), IRH (Intraradical hyphae), Ves (vesicle) and Arb (arbuscule)

Mycorrhizal colonization of *V. faba* roots was successful under all AM-inoculated treatments (Table 1 and Fig. 1). Under well-watered conditions, mycorrhizal colonization was robust, with fungal hyphal colonization (HC%) of 91.33 ± 4.83% and arbuscules (ARC%) and vesicles (VC%) percentages were 30.11 ± 1.59% and 74.25 ± 3.92%. Although drought stress negatively affects mycorrhizal colonization in *V. faba* roots, colonization is not completely inhibited. Drought stress significantly reduced colonization rates, with HC% dropping to 75.06 ± 3.97% and ARC to 16.66 ± 0.88%, suggesting an overall decline in symbiotic efficiency. Despite this reduction, colonization was still evident, indicating that mycorrhizal fungi retain some colonization ability even under water-limited conditions. Also, AM fungi can still confer benefits to host plants, albeit at a reduced level which indicates that drought can inhibit mycorrhizal development, but some fungal species can adapt and maintain symbiosis to a certain extent.

**Table 1:** Mycorrhization levels in *Vicia faba* L. plants under the effect of drought stress (DS: 30%WHC)

Treatments/ parameters	Hyphal colonization (HC)	Vesicular colonization (VC)	Arbuscular colonization (ARC)
Control	0c	0c	0c
DS	0c	0c	0c
KN	0c	0c	0c
KN + DS	0c	0c	0c
AM	91.33 ± 4.832a	74.25 ± 3.928a	30.11 ± 1.593a
AM + DS	75.06 ± 3.971b	66.66 ± 3.527b	16.66 ± 0.881b

Data are means ± standard errors, means followed for the same letters in the columns do not differ statistically by Duncan test, *p* < 0.05

In drought-stressed plants, a shift in colonization structures was observed as revealed in a recent study on soybeans [13]. Although hyphal penetration remained relatively stable, the formation of vesicles and arbuscules decreased significantly as arbuscules are vital for nutrient exchange and are particularly sensitive to carbon limitation and water deficit [46]. This may be due to impaired carbohydrate allocation to the fungi as drought limits photosynthesis, reducing the carbon supply to the fungal symbiont [47]. As well, Huang et al. [48] also stated that AM fungal spore germination decreased under drought stress which resulted in a notable reduction in the production of novel endogenous fungal structures. The level of colonization under drought stress also depends on the fungal species used.

In this study, drought-stressed plants showed reduced root branching and overall root surface area, limiting potential entry points for fungal colonization. Mycorrhizal fungi typically rely on healthy root development for effective colonization. Consequently, the stress-induced reduction in fine root structures may partially explain the decreased colonization frequency [49, 50]. Additionally, drought stress may increase the deposition of lignin and suberin in root cortical tissues [51, 52], acting as a physical barrier to fungal entry.

#### Plant growth and biomass

**Fig. 2:**
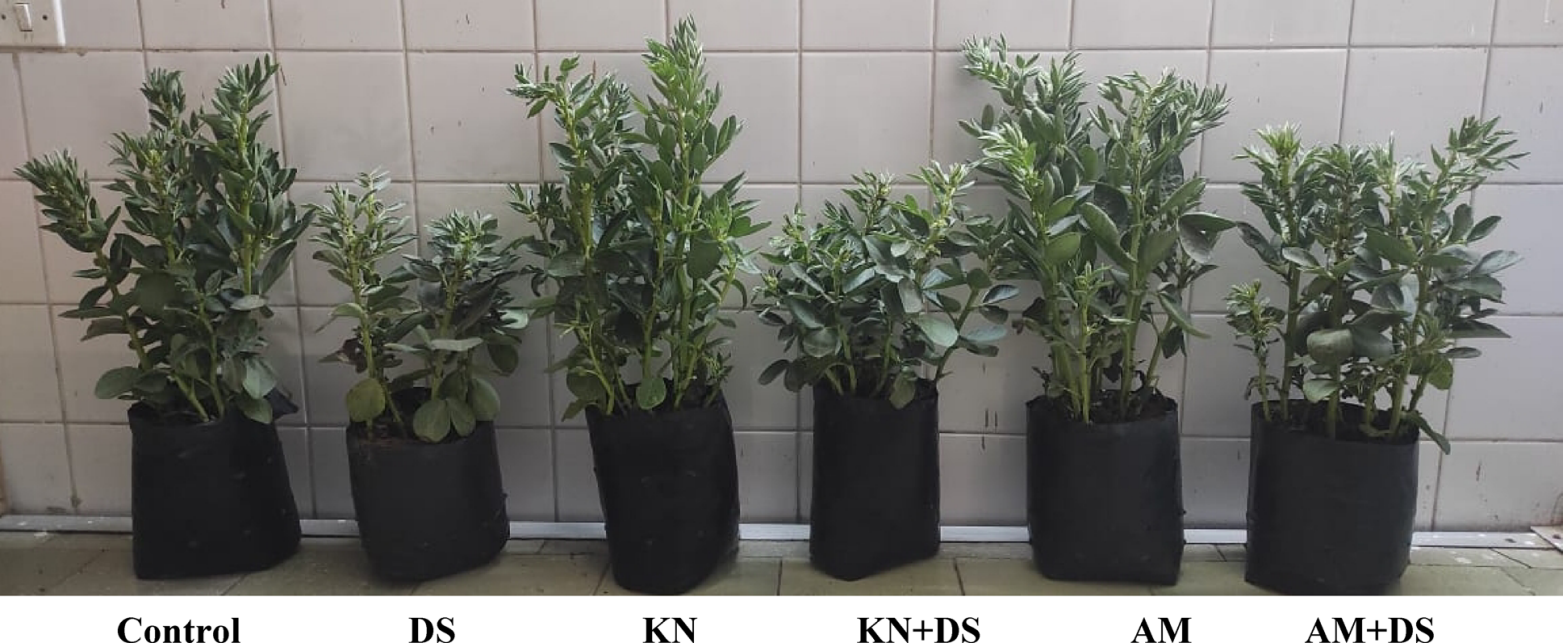
The performance of *V. faba* L. plants exposed to normal irrigation (Control) and drought stress (DS: 30% WHC) under the effect of kinetin (KN) and arbuscular mycorrhiza (AM)

**Fig. 3:**
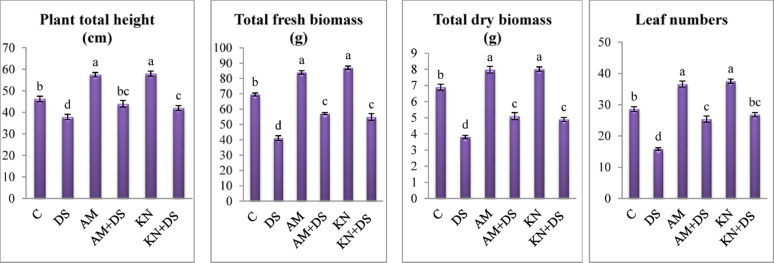
Comparison of statistical significance of differences in *V. faba* L. growth parameters across the influence of kinetin (KN) and arbuscular mycorrhiza (AM) under drought stress (DS: 30% WHC). Data are means ± standard errors, columns followed by the same letters do not differ statistically by Duncan, *p* < 0.05

Results in Figs. 2 and 3 revealed that drought stress (30% WHC) significantly impaired the vegetative growth of *V. faba*, as evidenced by reductions in plant height, fresh and dry biomass, and leaf numbers. Compared to the control (90% WHC; *p* < 0.05), drought-stressed plants exhibited a 40.8 and 44.8% reduction in *V. faba* fresh and dry biomass. Plant height and leaf numbers were reduced by 18.2% and 44.7%, respectively. These results are consistent with previous studies on faba bean, malva and soybean plants under drought stress [8, 13, 53] as this stress severely restricts plant growth by disrupting water relations, reducing photosynthesis, and impairing nutrient uptake [7, 54]. Also, this stress severely restricts cell expansion and induces oxidative damage [8, 55] and increases the synthesis of senescence-associated genes that code for cysteine proteases which promote early senescence of leaves and flowers in plants [56].

However, kinetin-treated plants (20 mg/L) showed improvement in plant fresh and dry biomass (33.39 and 28.68%, respectively as compared to drought-stressed plants without kinetin) and also enhanced leaf number (Fig. 3). The increases in plant height and fresh and dry biomass in *V. faba* align with Khurshid et al. [12] and Akter et al. [57] results. This improvement is due to the kinetin’s ability to delay leaf senescence, promote cell division, and enhance antioxidant defense mechanisms [58, 59]. A study by Rafiqul Islam et al. [17] revealed that the administration of exogenous kinetin increased endogenous kinetin levels, which in turn improved plants’ ability to overcome adverse growth impacts caused by water scarcity.

Similarly, AM-treated plants enhanced drought resilience where, under drought, it exhibited 38.76 and 34.2% greater fresh and dry biomass than non-AM, drought-stressed counterparts (*p* < 0.05). As well leaf number and plant height improved by 60.7 and 16.3%, respectively (Fig. 3). AM fungi are known to improve plant drought tolerance by improving root biomass and architecture, enhancing water uptake, cycling nutrients and improving soil structure [13, 24]. Through its hyphae, AM fungi improves nutrient uptake, especially N, P, and K, as well as water absorption [60, 61]. This is because the mycelium can spread and grow outside the rhizosphere and increase the root surface allowing the root area to absorb more nutrients that are immobile and rarely reach the plant’s roots [27, 62]. Also, this symbiotic association induces systemic changes in host plants, including improved antioxidant activity and osmolyte accumulation (shown later), which help maintain cellular homeostasis under drought conditions [63]. These physiological benefits likely contributed to the improved growth of mycorrhiza-treated *V. faba* plants under drought.

#### Photosynthetic pigments

**Fig. 4:**
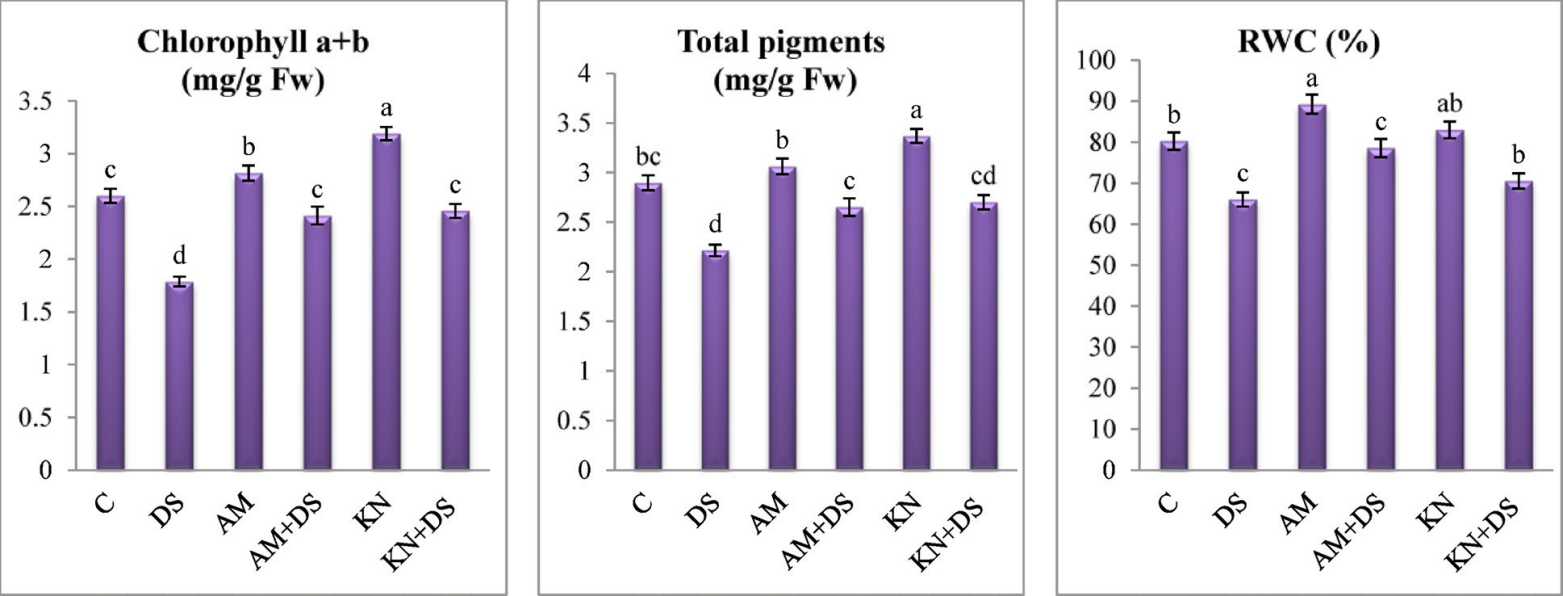
One-way ANOVA for determining differences between means for chlorophyll a + b and total pigments and relative water content (RWC) in *V. faba* L. plants across the influence of kinetin (KN) and arbuscular mycorrhiza (AM) under drought stress (DS: 30% WHC). Data are means ± standard errors, columns followed by the same letters do not differ statistically by Duncan test, *p* < 0.05

Chlorophyll is the green pigment present in chloroplasts in which photosynthesis takes place, thus it is crucial to determine chlorophyll and other pigments under the different treatments (drought, kinetin and AM). Compared to the well-watered treatment (Fig. 4), the photosynthetic pigments showed steadily declining values throughout drought; it reduced total chlorophyll (a + b) and total pigments levels by 31.5 and 23.7% respectively. Similar outcomes under drought stress were noted in *Avena nuda* [64] and *Malva parviflora* [8] plants. The decline in chlorophyll under water-deficient conditions is attributed to pigment degradation, inhibition of chlorophyll biosynthesis, and increased oxidative damage. Also, by increasing the activity of chlorophyllase and decreasing the activity of other associated enzymes in the synthesis of chlorophyll, drought stress degrades pigments [65, 66]. According to Rafiqul Islam et al. [17] and Vitale et al. [67], stomatal closure and subsequently a decrease in photosynthetic capacity seemed to be the main causes of drought-stressed plants’ decreased photosynthesis. To enable a higher rate of photosynthesis, a higher CO_2_ fixation per unit of leaf area requires high stomatal conductance. The stomata stay closed for a long period during drought conditions, which lowers CO_2_ absorption and water loss to maintain plant water status [17].

Furthermore, kinetin and AM treatment ameliorated this decline and maintained significantly higher chlorophyll levels compared to untreated counterparts. Both treatments significantly restored chlorophyll content, enhancing total chlorophyll (a + b) by 37.6 and 35.1% and total pigments by 22.2 and 19.9%. Kinetin counteracted drought effects by delaying senescence and maintaining photosynthetic machinery. This is attributed to kinetin’s ability to stabilize chloroplast membranes and promote chlorophyll biosynthesis [68] and enhance antioxidant enzyme activity, thereby reducing oxidative degradation of pigments [69, 70] that ultimately supports better photosynthetic efficiency under water-deficit conditions.

Likewise, during water stress, AM fungi increased the amount of chlorophyll and stomatal conductance compared to non-mycorrhizal ones under controlled and stressed conditions [71, 72]. Hashem et al. [50] stated that under drought-stressed and non-stressed conditions, AM-inoculated plants boosted pigment synthesis by decreasing chlorophyllase activity and upregulating the gene expression involved in chlorophyll biosynthesis.

#### Relative water content

The improved crop management in drought-prone areas depends on understanding the existing condition of water relations. RWC is a crucial feature among a number of water-related measures that are employed as an indicator for dehydration tolerance [73]. Drought-induced reductions in RWC led to turgor loss, which in turn reduced the amount of water available for plant cell division [17, 74]. We found that exposure of *V. faba* plants to drought stress (30% WHC) led to a significant decline in RWC which dropped significantly by 17.76% (Fig. 4), compared to well-watered controls (90% WHC) indicating water loss and reduced turgor. This reduction is a typical physiological response to water deficit and is indicative of the plant’s impaired ability to maintain turgor pressure and metabolic activity under stress [7, 75]. Due to the detrimental effects of drought on stomatal opening and closure processes, drought stress raises leaf temperature and decreases water availability, transpiration rate, and leaf water potential, all of which have an impact on plant water status [76].

It is worth noting that foliar-sprayed kinetin or mycorrhizal inoculation under drought stress significantly improved RWC in *V. faba* leaves by 7 and 19.1% compared to untreated stressed plants (Fig. 4), suggesting improved water retention and osmotic adjustment. Kinetin is known to delay senescence, and enhance osmolyte accumulation, all of which contribute to better hydration status under drought [58]. The observed increases in RWC in *V. faba* with kinetin application are consistent with Rafiqul Islam et al. [17] in maize plants suggesting that kinetin application assisted plants in maintaining their water status for improved growth. According to Malinowska et al. [77] reports, shoot-driven kinetin migrated basipetal towards the roots, causing the root system to grow so that it could forage for more water-absorbing space when there was a dearth of water. Therefore, plants can maintain better water status and flourish under water deficit by using kinetin-induced modulation of root structure and growth.

The alleviating of drought adverse effects on RWC by AM fungal inoculation in *V. faba* plants may be likely due to the improved root hydraulic conductivity and increased water uptake efficiency via the extended hyphal network [63, 78]. Our results are in line with those of Soliman et al. [13] and Oliveira et al. [79], who found that the water potential of soybean plants treated with AM fungi increased under water deficient circumstances. AM fungi also trigger physiological changes in host plants that improve osmotic adjustment and promote stomatal regulation, further contributing to improved hydration. The increase in RWC and biomass aligns with AM-induced upregulation of aquaporins and stress-responsive genes [63].

#### Stress markers (EL and H_2_O_2_)

Drought stress significantly increases EL and H_2_O_2_ in *V. faba* leaves, indicating compromised cell membrane integrity (Table 2). Under drought, EL of faba bean escalated from approximately 21.31% under well-watered conditions to over 51%. Quantitatively, H_2_O_2_ content provoked by 23.4% in drought-stressed plants relative to non-stressed controls (*p <* 0.05), showed the highest H_2_O_2_ content.

**Table 2:** One-way ANOVA for determining differences between means of stress markers in *V. faba* L. plants across the influence of kinetin (KN) and arbuscular mycorrhiza (AM) under drought stress (DS: 30% WHC)

Treatments/ parameters	H_2_O_2_ (mg/g Fw)	El (%)
Control	9.74 ± 0.257bc	26.028 ± 0.688c
DS	12.02 ± 0.318a	51.09 ± 1.352a
AM	9.483 ± 0.25c	21.308 ± 0.564d
KN	9.189 ± 0.243c	16.508 ± 0.437e
AM + DS	10.51 ± 0.278b	31.708 ± 0.838b
KN + DS	10.44 ± 0.276b	17.178 ± 0.45e

Data are means ± standard errors, means followed for the same letters in the columns do not differ statistically by Duncan test, *p* < 0.05

The increase in EL correlated with elevated H_2_O_2_ levels (a marker of oxidative stress). The surge in H_2_O_2_ reflects impaired ROS scavenging mechanisms and heightened lipid peroxidation, as evidenced by elevated malondialdehyde in drought-stressed soybean plants [13]. These results corroborate findings in mung bean [80] and faba bean [81].

Foliar-sprayed kinetin (20 mg/L) reduced EL and H_2_O_2_ content by 66.37 and 13.14% relative to stressed plants (Table 3) (*p* < *0.05*), surpassing the mitigation effect of AM fungi. Kinetin enhances enzymatic antioxidants and non-enzymatic scavengers like ascorbate-glutathione pools, as reported in *Triticum aestivum* [82]. Additionally, kinetin-treated plants retained higher chlorophyll content (Fig. 4), indicating preserved photosynthetic machinery as a critical factor in minimizing electron leakage and ROS generation in chloroplasts. This aligns with Merewitz et al. [83], who attributed cytokinin-induced drought resilience to delayed senescence and sustained photochemical efficiency. The decrease in H_2_O_2_ could thus be related to the protective effects of kinetin on plant photosynthetic machinery and cellular membranes, which damaged by oxidative stress. Our results are also consistent with Rafiqul Islam et al. [17], who reported that kinetin effectively reduced EL in maize grown under drought stress due to the antioxidant and protective properties of cytokinins. In wheat plants subjected to PEG-induced drought stress, kinetin-mediated recovery from membrane damage and an increase in cell viability were documented [84].

**Table 3:** The percentages of the comparative effectiveness of kinetin (KN) vs. arbuscular mycorrhizal (AM) fungi in promoting *V. faba* L. plant growth under controlled and drought-stressed conditions based on measured morphological, physiological and biochemical parameters

Parameters	Control conditions		Drought stressed conditions	
	Kinetin (KN)	Arbuscular mycorrhiza (AM)	Kinetin (KN)	Arbuscular mycorrhiza (AM)
Plant height (cm)	25.59	24.29	11.05	16.34
Total fresh biomass (g)	25.10	20.69	33.39	38.74
Total dry biomass (g)	16.25	15.82	28.68	34.21
Chlorophyll a + b (mg/g Fw)	23.16	8.49	38.03	35.39
Total pigments (mg/g Fw)	16.29	5.88	22.17	19.90
RWC (%)	3.42	11.21	6.89	19.06
El (%)	− 36.57	− 18.13	− 66.37	− 65.33
H_2_O_2_ (mg/g Fw)	− 5 0.74	− 2.66	− 13.14	− 12.56
Proline (µM/g Fw)	− 25.78	12.43	19.95	90.44
Protein (mg/g Fw)	44.98	95.47	38.99	53.19
Carbohydrates (mg/g Dw)	24.29	41.21	7.54	28.64
Easily glomalin (mg/g Fw)	8.26	34.10	12.70	58.50
Total glomalin (mg/g Fw)	2.79	27.29	6.71	55.41
SOD (U/g Fw)	53.06	49.61	26.52	25.60
CAT (U/g Fw)	− 5.00	−1.25	10.00	5.76
POX (U/g Fw)	−1.96	−1.44	18.56	7.95

*RWC: relative water content, El: electrolyte leakage, SOD: superoxide dismutase, CAT: catalase, and POX: peroxidase

Furthermore, AM inoculation significantly attenuated EL and H_2_O_2_ under drought as compared to untreated stressed plants (*p* < *0.05*). These findings are mirror observations in *Glycine max*, where AM fungal colonization preserved membrane integrity under drought via ROS regulation [13, 85]. AM fungi enhances antioxidant enzyme activity, including CAT and POX, thereby improving ROS detoxification [63, 86]. Additionally, mycorrhizal plants exhibited improved root hydraulic conductivity and enhanced plant water and nutrient uptake, particularly phosphorus, which is essential for maintaining cellular membrane integrity [87]. AM fungi mitigated EL by enhancing osmotic adjustment *via* glomalin-related soil proteins and upregulating antioxidants. Lower H_2_O_2_ levels correlate with improved membrane stability and higher biomass in treated plants, suggesting preserved cellular function of kinetin application or AM fungal inoculation under stress.

#### Osmotic and organic adjustment

**Fig. 5:**
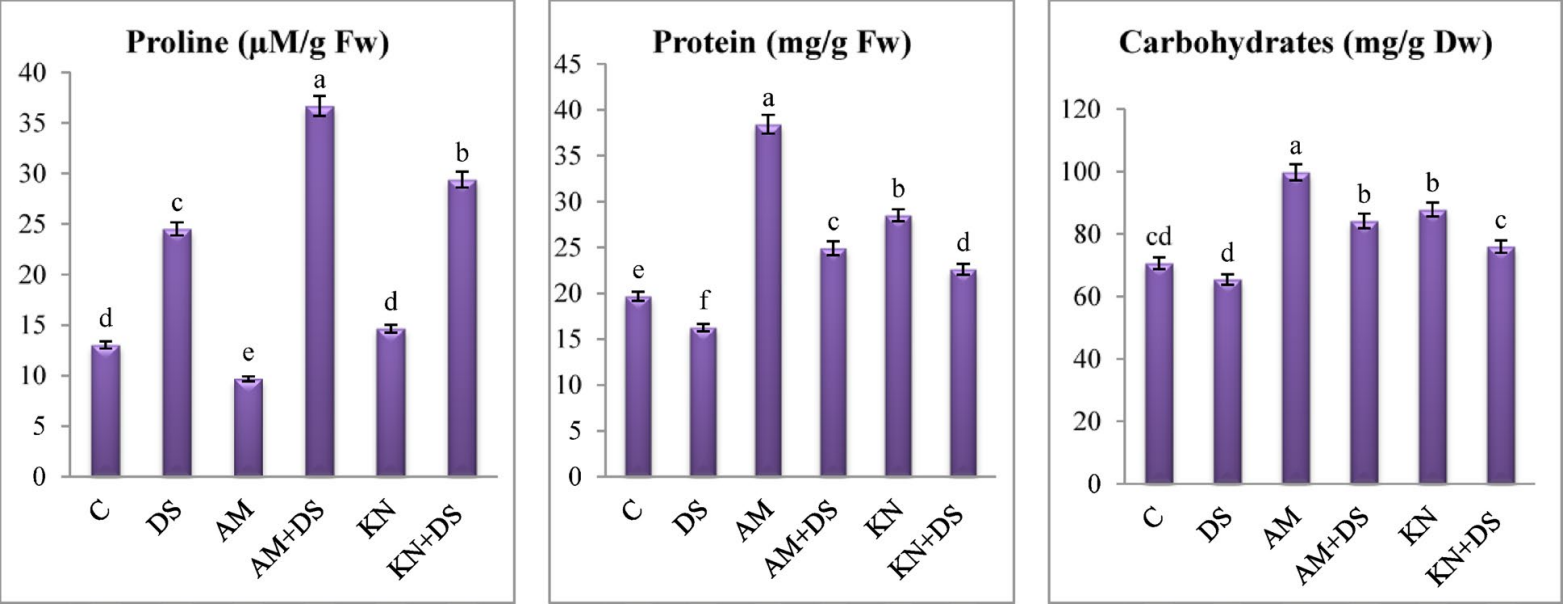
One-way ANOVA for determining differences between means for osmotic substances in *V. faba* L. plants across the influence of kinetin (KN) and arbuscular mycorrhiza (AM) under drought stress (DS: 30% WHC). Data are means ± standard errors, columns followed by the same letters do not differ statistically by Duncan test, *p* < 0.05

Plants use a variety of physiological and biochemical responses such as osmoprotectant to defend against water-deficit stress. The most key osmoprotectants are proline, protein and soluble sugars [88]. In our investigation, proline, carbohydrates and soluble protein are affected by drought (30% WHC), kinetin (20 mg/L) and AM inoculation. The results demonstrated a significant increase in proline content in *V. faba* plants subjected to drought stress compared to well-watered controls (Fig. 5). Drought-stressed plants exhibited a two-fold increase in proline levels relative to non-stressed controls, reflecting an adaptive osmoprotective response and membrane protection [89].

Application of kinetin or AM inoculation further enhanced proline accumulation under drought conditions. The drought + kinetin treatment resulted in a more than two-fold increase in proline compared to the non-stressed control, while drought + AM plants showed a three-fold increase. These findings suggest that both kinetin and mycorrhizal colonization enhance the osmotic adjustment capacity of *V. faba* under drought stress. Proline is well-established as a compatible solute that stabilizes proteins and membranes and maintains cellular turgor under dehydration [90]. As a molecular chaperone, proline’s antioxidant qualities have been found to be useful as a ROS scavenger. The pyrroline-5-carboxylate reductase enzyme is highly expressed in stressed plants, which causes proline to build up [91].

Regarding carbohydrate, drought stress significantly affected its content in *V. faba* leaves as observed in Fig. 5. Compared to the well-watered control, stressed plants showed a 7.4% reduction in total soluble carbohydrates (*p* < 0.05), highlighting the adverse effects of water deficit on photosynthetic activity and carbon assimilation. Most obvious that kinetin and AM fungal inoculation under drought conditions led to a partial restoration of carbohydrate levels, registering 16.2 and 28.7% increase over the stressed untreated group. These findings underscore the capacity of both kinetin and AM fungi to alleviate drought-induced suppression of carbohydrate metabolism. The improvement in carbohydrate levels in AM-treated plants can be attributed to enhance water and mineral uptake, particularly phosphorus, which is critical for ATP production and photosynthetic efficiency [92]. Mycorrhizal colonization has also been shown to enhance stomatal regulation and photosynthetic pigment stability under water stress, leading to improved carbon fixation [13, 93]. In kinetin-treated plants, the improved carbohydrate status may reflect the hormone’s role in maintaining chloroplast integrity, thereby sustaining photosynthetic capacity during stress, delaying senescence-associated degradation processes and upregulating protein synthesis pathways through increased ribosomal activity and transcriptional stability [94].

The results presented in Fig. 5, indicate a significant decline in total protein content in stressed *V. faba* plants (16.3) compared to non-stressed controls (19.7 mg/g fresh weight). This decline is consistent with Farooq et al. [7] who indicated that drought impairs nitrogen metabolism and protein biosynthesis while promoting proteolysis. Under drought, kinetin application led to a moderate recovery in protein levels, increasing content by 38.9% relative to the drought-only **(**Table 3**)**. Probably, the increase in protein level may be induced by elevated and potentially prolonged expression of target genes in response to the addition of the hormone [18].

AM-inoculated plants showed a greater increase (53.3%) over drought controls (Table 3).The protein enhancement under AM fungal inoculation can be attributed to improved nutrient acquisition, particularly nitrogen, and the modulation of nitrogen-assimilating enzymes such as nitrate reductase and glutamine synthetase [60, 92]. AM fungal symbiosis also stabilizes cellular homeostasis, reducing proteolytic degradation and promoting biosynthetic pathways [93].

#### Easily and total glomalin production

**Fig. 6:**
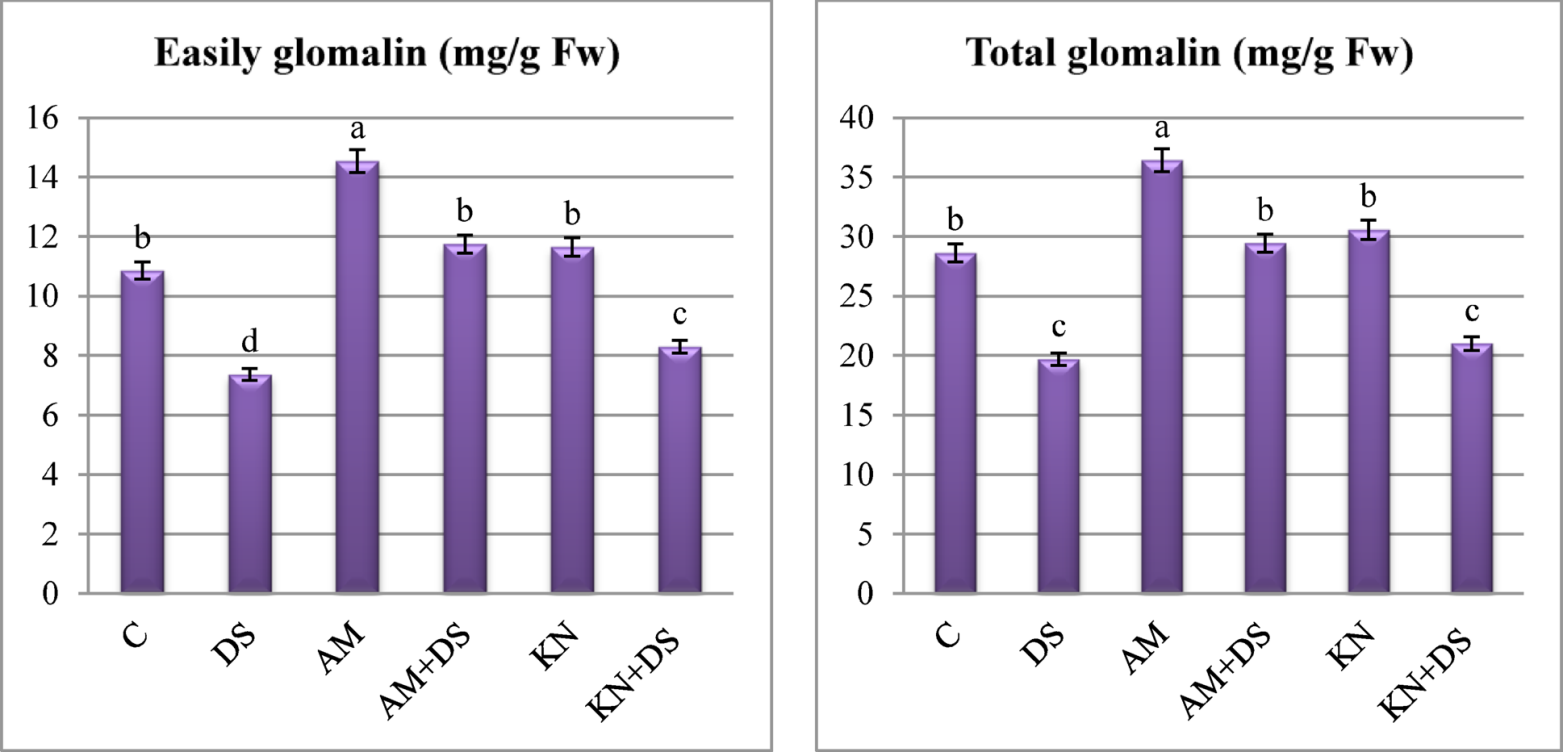
One-way ANOVA for determining differences between means for glomalin content in *V. faba* L. plants across the influence of kinetin (KN) and arbuscular mycorrhiza (AM) under drought stress (DS: 30% WHC). Data are means ± standard errors, columns followed by the same letters do not differ statistically by Duncan test, *p* < 0.05

Glomalin-related soil protein, a biomarker for AM fungal activity and soil aggregation, were markedly influenced by drought stress, mycorrhizal colonization (to greater extent) and kinetin (to lower extent). Non-inoculated *V. faba* plants under drought stress exhibited a significant decrease in its content compared to non-stressed controls **(**Fig. 6**)**, indicating impaired fungal functionality under water deficit. Compared to the well-watered control, drought resulted in a 32.2 and 31.3% reduction in EEG and TG contents, indicating an adverse impact of water limitation on glomalin secretion and microbial activity. However, exogenous application of kinetin under drought increased EEG and TG by 12.70 and 6.71% relative to the drought treatment (Table 3). A more substantial improvement was observed in AM-inoculated plants, where EEG and TG increased by 34.1 and 27.3% compared to controls. Interestingly, AM-inoculated plants under drought stress demonstrated a substantial increase in glomalin production, with EEG and TG levels were approximately 1.5 times higher than non-inoculated stressed plants (Fig. 6).

Glomalin-related soil proteins are crucial role in soil health, water retention, and plant stress tolerance. Secreted by AM hyphae, glomalin acts as a hydrophobic glycoprotein that promotes soil aggregation and stabilizes soil structure by binding microaggregates into macroaggregates [95]. This structural improvement enhances soil porosity and water-holding capacity, which is vital under drought conditions. The observed decrease in EEG and TG under drought indicates that water limitation suppresses AM fungal metabolic activity and consequently glomalin production [96]. However, AM-inoculated plants exhibited significantly higher levels of EEG and TG under drought, suggesting that mycorrhizal symbiosis remains partially active even under stress. Our results are in line with Soliman et al. [13] and Cheng et al. [97], who found that the presence of AM fungi raised the levels of both EEG and TG while drought stress decreased their contents, suggesting that drought inhibits the glomalin synthesis. Higher glomalin levels are also linked to improved microbial habitat stability and nutrient cycling, thus supporting a healthier rhizosphere and promoting plant survival under stress [95, 96]. The enhancement of glomalin levels with kinetin application, although less pronounced than with AM fungi, is notable. Kinetin may support glomalin production indirectly by maintaining root vigor and its known role in stabilizing cellular membranes and improving water status in stressed plants could translate into better support for microbial partners in the rhizosphere. These findings reinforce the ecological importance of AM-derived glomalin in drought-stressed systems and reveal an auxiliary role for cytokinins like kinetin in sustaining microbial-plant interactions.

#### Antioxidant enzyme activity

**Fig. 7:**
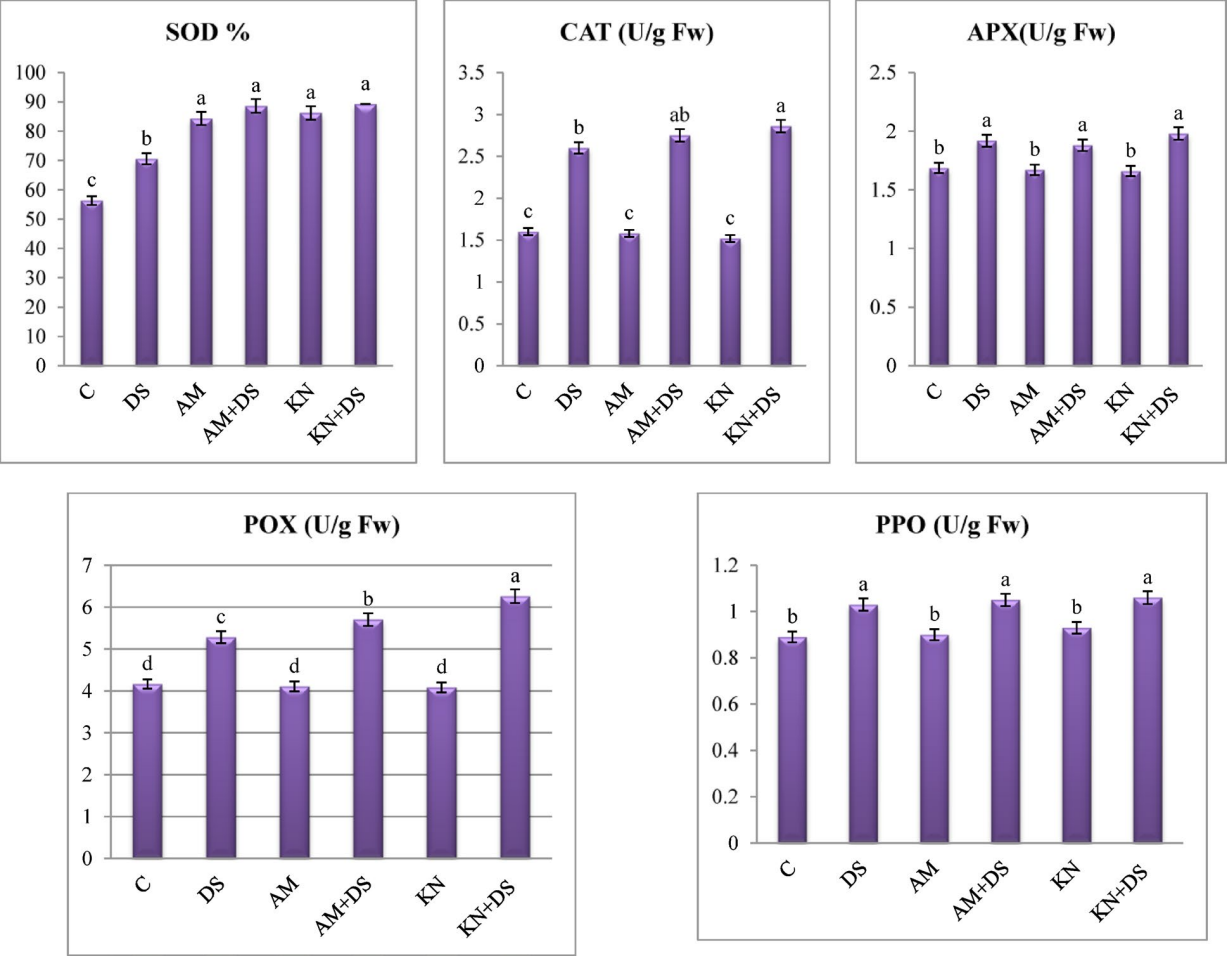
One-way ANOVA for determining differences between means of antioxidant activity (superoxide dismutase: SOD, catalase: CAT, ascorbate peroxidase: APX, peroxidase: POX and poly phenol oxidase: PPO) in *V. faba* L. plants across the influence of kinetin (KN) and arbuscular mycorrhiza (AM) under drought stress (DS: 30% WHC). Data are means ± standard errors, columns followed by the same letters do not differ statistically by Duncan test, *p* < 0.05

Drought stress significantly influenced the activity of antioxidant enzymes in *V. faba*, with notable alterations in SOD, CAT, PPO, APX and POX levels (Fig. 7). Enzyme assays revealed that drought induced a pronounced increase in enzyme activities relative to well-watered controls, reflecting a robust oxidative stress response. SOD activity increased by 25.2%, CAT by 62.5%, PPO by 15.7%, APX by13.8% and POX by 26.8% under drought stress compared to well-watered controls. These results align with El-Sappah et al. [98] and Hasanuzzaman et al. [99, 100] who noted that drought induces ROS scavenging antioxidative defense systems. Under drought stress, the increase in SOD activity enhanced the dismutation of superoxide radicals into H_2_O_2_. Similarly, CAT and POX activity reflects an upregulation of H_2_O_2_-scavenging pathways.

Application of kinetin significantly moderated the stress-induced elevations in enzyme activity. In drought-stressed plants treated with kinetin, SOD, CAT and POX activities were elevated significantly by 26.52% for SOD, 10.00% for CAT and 18.56% for POX (Table 3), while a non-significant change was observed in the activities of APX and PPO relative to the drought stressed plants (Fig. 7). This result suggests that kinetin plays a regulatory role in modulating ROS levels by improving cellular water status and membrane stability, thereby reducing oxidative pressure [101]. Similarly, according to the findings of Chang et al. [102], exogenous kinetin may boost antioxidant enzyme activity and strengthen creeping bentgrass’s antioxidant defense against drought stress. Kinetin treatment in sesame plants under drought stress resulted an upsurge in POX, suggesting its role in suppressing the formation of harmful hydroxyl radicals [12, 103]. Additionally, treatment with zeatin riboside [104] or dihydrozeatin [105] was found to increase CAT and SOD activity. El-Badri et al. [106] demonstrated kinetin’s role in upregulating *Cu/Zn-SOD* and *CAT2* genes in drought-stressed maize. Kinetin likely preserves enzyme functionality by maintaining cellular hydration and activating stress-signaling pathways, such as mitogen-activated protein kinases (MAPKs), which trigger antioxidant biosynthesis.

**Fig. 8:**
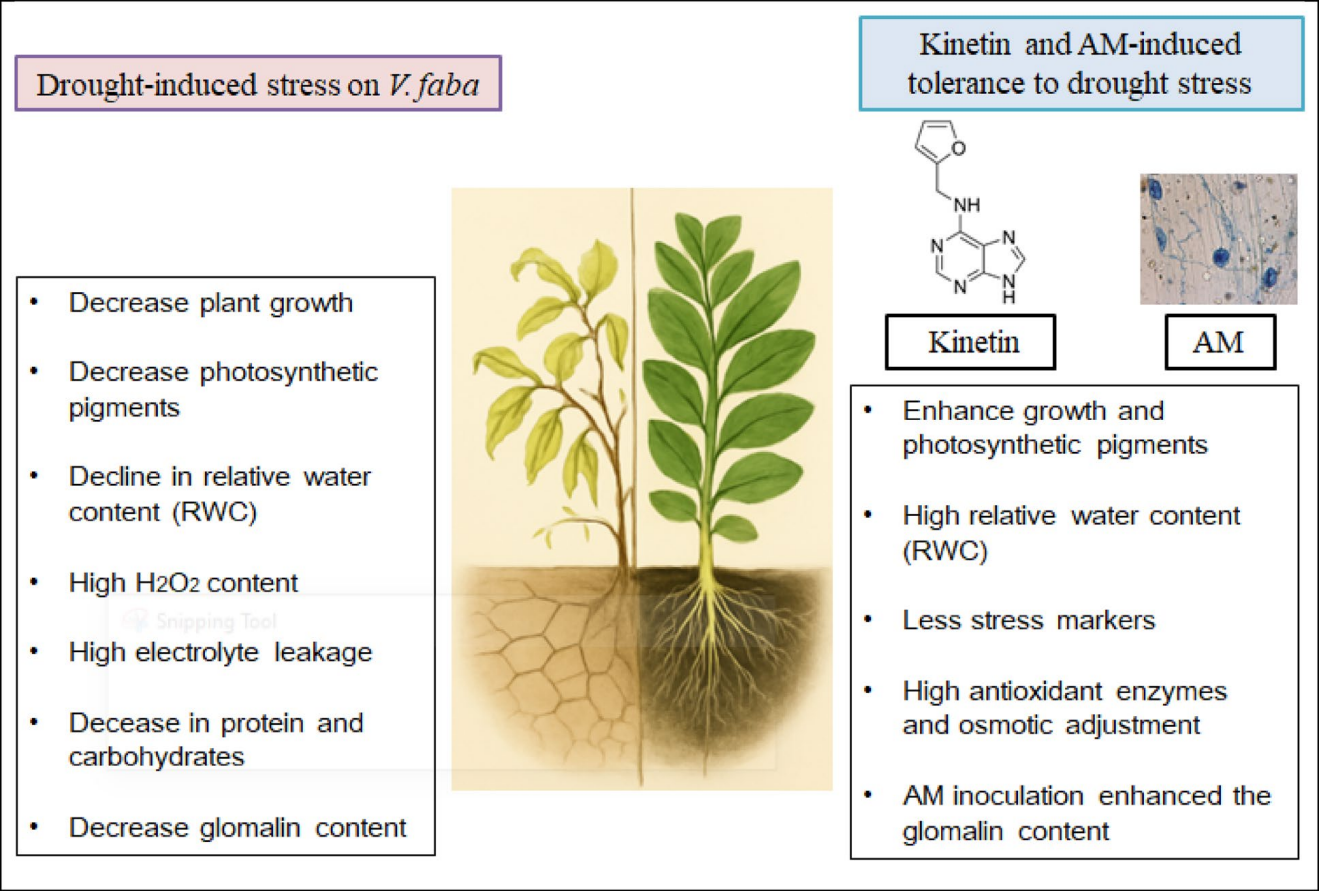
Illustrative overview for the effect of drought on *V. faba *plants and the role of kinetin or AM fungi in regulating the drought tolerance

Inoculation of *V. faba* with AM fungi also conferred significant mitigation of oxidative stress by exhibiting increases of 25.60% (SOD), 5.76% (CAT), and 7.95% (POX) (Table 3) in AM-treated drought-stressed plants over drought-stressed only (Fig. 7). These results indicate that AM fungi enhances antioxidant defense, possibly through enhanced water absorption and priming of systemic defense responses. Recent studies affirm that AM fungal symbiosis can modulate redox homeostasis and bolster drought resilience in legumes through antioxidant activation and improved physiological status [85, 107]. Collectively, an illustrative overview for the effect of drought on *V. faba* plants and the role of kinetin or AM in regulating the drought tolerance by preserving the morphological and physiological characteristics, antioxidant enzyme activity and osmolytes synthesis was represented in Fig. 8.

## Conclusion

The study demonstrates that drought stress in *V. faba* significantly impairs physiological and biochemical functions. However, exogenous kinetin and inoculation with endophytic mycorrhizal fungi independently improved its tolerance. These improvements are mediated *via* better growth, water retention, enhanced antioxidant defenses, and maintenance of photosynthetic pigments. Integrating kinetin and endophytic mycorrhizal fungi offers a promising strategy for enhancing drought resilience under climate-induced water scarcity in *V. faba* and could serve as a sustainable strategy to improve crop productivity and may be practically implemented in arid and semi-arid agricultural systems to mitigate drought-induced yield losses. Future research should focus on identifying specific fungal strains and optimal application protocols for field conditions. Understanding the molecular interactions between kinetin signaling and fungal symbiosis will pave the way for sustainable agricultural practices.

## Data Availability

Data is provided within the manuscript.

## Abbreviations

ARC: Arbuscular colonization
AM: Arbuscular mycorrrhizal
APX: Ascorbate peroxidase
CAT: Catalase
EEG: Easily extractable glomalin
$$\text{H}_2\text{O}_2$$: Hydrogen peroxide
HC: Hyphal colonization
POX: Peroxidase
PPO: Polyphenol oxidase
ROS: Reactive oxygen species
RWC: Relative water content
SOD: Superoxide dismutase
TG: Total glomalin
VC: Vesicular colonization
WHC: Water holding capacity

## Declarations

### Experimental research and field studies on plants

“All relevant institutional, national and international guidelines and legislation were compiled or adhered to in the production of this study”.
